# The Pharmacological Activities of (−)-Anonaine

**DOI:** 10.3390/molecules18078257

**Published:** 2013-07-12

**Authors:** Hsing-Tan Li, Hui-Ming Wu, Hsin-Liang Chen, Chi-Ming Liu, Chung-Yi Chen

**Affiliations:** School of Medical and Health Sciences, Fooyin University, Kaohsiung 83102, Taiwan; E-Mails: htli9796@ms16.hinet.net (H.-T.L.); mt019@fy.edu.tw (H.-M.W.); percy283@yahoo.com.tw (H.-L.C.)

**Keywords:** (−)-anonaine, magnoliaceae, annonaceae

## Abstract

Several species of Magnoliaceae and Annonaceae are used in Traditional Chinese Medicine. (−)-Anonaine, isolated from several species of Magnoliaceae and Annonaceae, presents antiplasmodial, antibacterial, antifungal, antioxidation, anticancer, antidepression, and vasorelaxant activity. This article provides an overview of the pharmacological functions of (−)-anonaine.

## 1. Introduction

**Figure 1:**
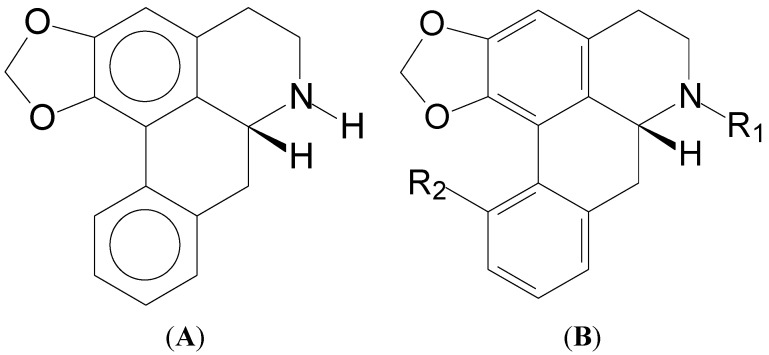
Chemical structure of (−)-anonaine (**A**) and anonaine derivative (B).

Various constituents of *Michelia alba* (Magnoliaceae) are used for medical purposes. In our research, we have identified a series of compounds from *Michelia alba*, including (−)-anonaine (Figure 1A) [1], an aporphine (isoquinoline) alkaloid also isolated from other plants (Table 1) with interesting and varied biological and pharmacological activities, including vasorelaxant, antibacterial, antifungal, antioxidative, anticancer and antidepressant effects, as summarized in Table 1 [1,2,3,4,5,6,7,8,9,10,11,12,13,14,15,16,17,18,19,20,21,22,23,24,25,26,27,28,29,30,31,32]. In this article, we will focus on describing the pharmacological mechanisms of action of (−)-anonaine.

## 2. Anticancer Activity

There are three different mechanisms by which a cell commits suicide by apoptosis. One is initiated by signals arising within the cells. The second is triggered by death activators binding to receptors at the cell surface. The third is triggered by reactive oxygen species (ROS).

**Scheme 1:**
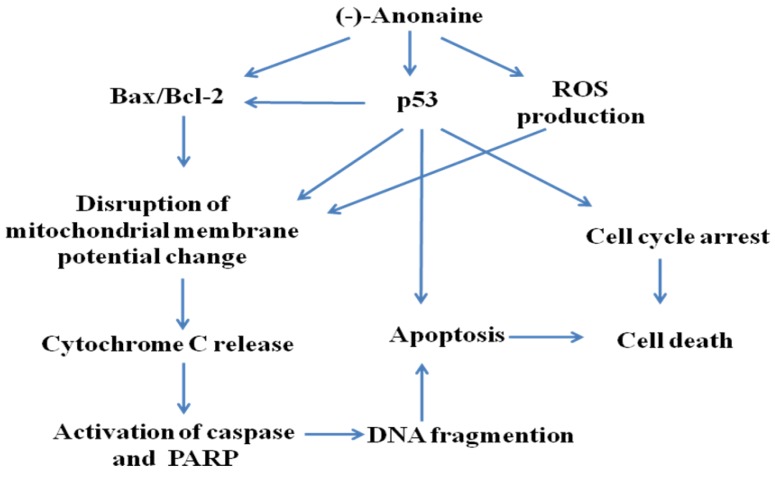
The possible mechanism of action of (−)-anonaine-induced apoptosis and cell cycle arrest in cancer cells.

Studies have demonstrated that (−)-anonaine has anticancer activity and cytotoxic effects in different cancer cell lines [6,7,15]. The viability of cells treated with (−)-anonaine decreased in a dose-dependent manner on different cell lines including HeLa, HepG2, rat hepatocytes, and H1299 [6,7,15]. In human cervical cancer cell (HeLa), (−)-anonaine caused DNA damage associated with increased intracellular nitric oxide, ROS, glutathione (GSH) depletion, disruptive mitochondrial transmembrane potential, activation caspase 3, 7, 8 & 9 activation and poly ADP ribose polymerase cleavage [15]. Moreover, (−)-anonaine also up-regulates the protein expression of p53 and Bax [15]. (−)-Anonaine is a potential anticancer agent against HepG2 (human liver carcinoma cell), rat hepatocyte with IC_50_ values of 33.5, 70.3 μg/mL [7]. This compound also exhibits antiproliferation, antimigratory effects, DNA damage and cell cycle arrest in human lung cancer cell (H1299) [6]. The above-mentioned results indicate that (−)-anonaine has cytotoxic activity (Scheme 1).

## 3. Vasorelaxation Activity

Reports have shown that aporphine alkaloids display a variety of different pharmacological activites in cardiovascular system [16,20,26]. (−)-Anonaine has Ca^2+^ channel blocking activity through voltage-operated channel and α_1_-adrenoceptor blocking activity in isolated rat thoracic aorta [20]. Recently, a study has shown that the affinities of (−)-anonaine for α_1_-adrenoceptor subtypes are in the order α_1A_＞α_1D_＞α_1B_ without inhibition phosphodiesterase enzymatic activity [16]. Further, this study confirms that α_1_-adrenoceptor subtypes selectivity of aporphine alkaloids can be modulated by the position of free hydroxyl (R2) and N-methyl (R1) substituents on the aporphine structure (Figure 1B) [16].

## 4. Antioxidative Activity

Oxidative stress is an imbalance of prooxidants and antioxidants in the organism. The oxidative stress can contribute to inflammation, heart disease, hypertension, various neurodegenerative diseases, and cancers. Anti-oxidative capacity of anonaine has been studied as a potential inhibitor of lipid peroxidation stimulated by Fe^2+^/cysteine in rat liver microsomal fractions [25]. The antioxidation activity of anonaine was also evaulated monitoring inhibition of microsomal lipid peroxidation induced by Fe^2+^/ascorbate, CCl_4_/NADPH or Fe^3+^ ADP/NADPH [21]. However, one study demonstrated that anonaine increased deoxyribose degradation by generated hydroxyl radical. This effect was determined by thiobarbituric acid method in the incubation medium Fe^3+^-EDTA and H_2_O_2_ [22]. 

## 5. Central Nervous System (CNS) Activity

Depression is a common mental disorder all over the World. Several species of Annonaceae are used in traditional medicine because of their anti-anxiety, anticonvulsant, and tranquilizing properties [2]. Previous study has shown that (−)-anonaine has good selectivity for ^3^H-dopamine uptake. The affinity of (−)-anonaine at dopamine D_1_^3^H-SCH 23390 and D_2_^3^H-raclopride binding sites was low [19]. (−)-Anonaine displays dopamine uptake inhibitory properties. 5-HT_1A_ receptor plays an important role in depressive disorders. One study has shown that 1,2-dimethoxy-5,6,6a,7-tetrahydro-4H-dibenzoquinoline-3,8,9,10-tetraol, (−)-anonaine, liriodenine, and nornuciferine are the main constituents of the aerial parts of *Annona cherimola* [2]. These main constituents produced antidepression-like effects due to the 5-HT_1A_ receptor agonistic activity of (−)-anonaine and nornuciferine [2]. These results indicate that (−)-anonaine displays dopamine uptake inhibitory and 5-HT_1A_ agonistic activity with anti-depressant activity.

Another study reported that (−)-anonaine at 0.05 μM reduced tyrosine hydroxylase (TH) and aromatic L-amino acid decarboxylase (AADC) activity [13]. In addition, (−)-anonaine at 0.05 μM reduced L-DOPA (50 μM and 100 μM)-induced increase in dopamine content without enhancing L-DOPA-induced cell death in PC12 cells at 24 h [13]. 

## 6. Antiparasitic Activity and Antimicrobial Activity

*Plasmodium falciparum* is the cause of malaria, a life-threatening disease for thousands of years all around the World, particularly in Africa. The drug resistance is reducing the therapeutic efficiency for the treatment malarial and parasite. The *in vitro* antiplasmodial activity of (−)-anonaine was examined [5,32]. One study has reported that (−)-anonaine has antiplasmodial activity against both chloroquine sensitive D10 strain (IC_50_ values of 25.9 ± 0.2 μM) and chloroquine resistant D12 strain of *Plasmodium falciparum* (IC_50_ values of 19.6 ± 1.1 μM) with low cytotoxicity in a Chinese Hamster Ovarian cell line (CHO) [5]. Another study indicated that (−)-anonaine has antiplasmodial activity by *in vitro* radiometric *Plasmodium falciparum* growth inhibition assay (IC_50_ values of 7 ± 2 μM) [32].

The antimicrobial effects of (+)-anonaine have been described in several studies, however, the exact mechanism of action remains unclear [24,27,28]. Studies have shown that (+)-anonaine has strong inhibitory activities against *Bacillus cereus*, *Escherichia coli*, *Micrococcus* sp., *Staphylococcus aureus* and *S. epidermidis* and displays anti-fungus activities against *Trichophyton rubrum* and *Microsporum gypseum* growth [24,27,28].

## 7. Conclusions 

With the current information, it is evident that anonaine has interesting pharmacological functions, including vasorelaxant, antbacterial, antifungal, antioxidative, anticancer and antidepressant effects. In addition, one approved U.S. patent reports that anonaine also has utility in the prevention and treatment of gastrointestinal dyskinetic diseases (US patent number US7198804) [33]. However, there is lack of correlation between *in vitro* and *in vivo* studies on the effects of anonaine Toxicity studies are missing too. For this reason, extensive pharmacological, chemical experiments and metabolism studies should be undertaken. Last but not least, this article aims to provide useful information about anonaine for researchers in this field.

## Figures and Tables

**Table 1:** Anonaine isolated from plants and its pharmacological effects.

Source	Pharmacological effects
Goniothalamus australis aerial parts of *Annona cherimola* bark of *Annona salzmannii* Michelia champaca Liriodendron tulipifera leaves of *Michlia alba* Magnolia grandiflora Stephania yunnanensis bark of *Guatteria hispida* Fissistigma latifolium Polyalthia longifolia Rollinia leptopetala fruit of *Annona muricata* Annona cherimolia Annona squamosa Artabotrys maingayi Chamanthera dependens Nelumbo lutea	antoxidant activity antidepression anticancer activity vasorelaxant activity antiplasmodial activity antibacterial activity antifungal activity
